# Measurements of traditional Chinese medicine health literacy regarding chronic pain: a scoping review

**DOI:** 10.1186/s12906-024-04698-6

**Published:** 2024-11-13

**Authors:** Zhiyi Qian, Grace Y Wang, Marcus Henning, Yan Chen

**Affiliations:** 1 Centre for Medical and Health Sciences Education, School of Medicine, University of Auckland Grafton Campus, 22-30 Park Ave, Grafton, Auckland New Zealand; 2 School of Psychology and Wellbeing, University of Southern Queensland, Ipswich, Australia; 3 Centre for Health Research, University of Southern Queensland, Ipswich, Australia

**Keywords:** Health literacy, Measurement, Traditional Chinese medicine, Chronic pain, Psychometric evaluation

## Abstract

Chronic pain is a prevalent health condition that imposes a significant burden on the global health system. Health literacy is a determinant of the quality of pain management which directly impacts public and individual health. However, the existing health literacy measurements have predominantly focused on medical models stemmed from Western culture and the knowledge of non-Western health models has largely been neglected. This review scopes refereed health literacy publications with regard to traditional Chinese medicine and chronic pain to explore and identify 1) the conceptual basis underlying the development of traditional Chinese medicine health literacy in this area, and 2) measurement tools used in this area and their associated psychometric qualities. Twenty-eight journal articles were assessed and the results showed that most studies’ conceptual frameworks were unable to cover three key health literacy aspects defined by the World Health Organization (access, understand, and apply). Furthermore, the identified health literacy measurement tools generally lacked rigorous psychometric evaluation. Future studies should focus on exploring a comprehensive model that encompasses various health models and developing measurement tools with more culturally representative psychometric assessments.

Chronic pain has been defined as pain that persists for an extended period (i.e., at least three months or longer) and lasts longer than the expected recovery time, which is a common complaint among patients with musculoskeletal diseases such as knee osteoarthritis and lumbar disc herniation [1]. Chronic pain has high prevalence among older adults and places a significant burden on the health system globally [2, 3]. Chronic pain can negatively affect persons’ daily function, job opportunities, psychological wellbeing, and quality of life [4–6]. Also, the recurring pain increases the chance of medication misuse which may cause addictive or other adverse medical issues [7]. Extensive clinical efforts have been made to alleviate chronic pain in individuals, including conventional methods (e.g., various types of analgesics or surgery), as well as complementary and alternative medicine (e.g., Chinese medicine or medication) [8, 9]. In addition, self-management, regarded as a critical skill for achieving better health outcomes in populations with chronic pain, is addressed in both clinical and community settings [10].

Health literacy (HL) is a concept initially proposed in the 1970s for western medicine [11] and has been widely accepted as one of the most influential determinants for both individual and public health [12]. According to the World Health Organization [13], health literacy significantly contributes to an individual’s capacity to improve their lifestyle and health status, which includes basic skills such as reading pamphlets and making appointments, and advanced abilities related to acquiring, understanding, and utilizing health information or services to make constructive health-related decisions [14]. Evidence suggests that health literacy is associated with the ability to manage pain and to improve health outcomes [15]. For example, a person with a low level of health literacy is likely to have less knowledge about medication and pain, behave poorly in regulating self-management, and, thus, suffer a higher intensity of pain.

When applied in the context of public health, the level of health literacy can be used as an evidence-based index to appraise and optimize health information, health services or health education programs, ultimately contributing to better health outcomes [16, 17]. Health literacy measurements with proven reliability and validity can help practitioners evaluate patients’ health-related knowledge and skill levels as well as identifying vulnerable populations [18]. At present, the most commonly used objective measurement tools of health literacy are the Newest Vital Sign (NVS) [19], the Rapid Estimate of Adult Literacy in Medicine (REALM) [20] and its revised versions (REALM- Short Form or REALM-Revised), and the Short Test of Functional HL in Adults (S-TOFHLA). There are also subjective tools such as the Single Item Literacy Screener (SILS) [21], Subjective Numeracy Scale [22], Brief HL Screen (BHLS) [23], and Chew et al.’s brief screener [24]. All these tools were developed based on Nutbeam’s [25] three-level model of health literacy that primarily focuses on the functional level (i.e., basic reading and writing skills to be able to understand and use health information). Further developmental work is needed to capture the more advanced aspects of health literacy, such as the ability to assess the validity of health claims.

In countries or regions with a strong Chinese cultural influence (i.e., Mainland China, Hongkong, Taiwan, Malaysia, Singapore), both traditional Chinese medicine (TCM) treatments (acupuncture, acupressure, massage, moxibustion, herbs, etc.) and western medicine treatments are used in the management of chronic pain. People who are accustomed to multiple health systems may hold different expectations and criteria when making health-related decisions. Their perceptions and understanding of intervention options influence the choice and effectiveness of treatment. For example, patients born and living in a country with the preference for western medicine tend to rely on biomedical options (i.e., physiotherapy, opioid medications, and surgery) to treat chronic pain. Conversely, those familiar with dual or multiple medical systems need to decide which system they should utilise, western medicine, TCM, or both. This decision-making process often results in various treatment options, each with their own therapeutic benefits and side-effects. Therefore, a comprehensive understanding of culturally specific health literacy is crucial for accurately and inclusively assessing health literacy and to ensure inclusivity among these groups [26, 27]. Failure to do so may result in barriers to health promotion and disease prevention [28].

Being aware of the importance of traditional Chinese medicine health literacy (TCM-HL), the Chinese government has published a guideline that consists of 66 items pertaining to the TCM preservation knowledge and skills which was proposed by TCM expert panels and believed to be beneficial to the general population in 2012 [29]. Several questionnaires have since been developed and applied in different regional groups [30–32]. According to the development procedure of these questionnaires, TCM-HL is defined as the ability of individuals to gain access to, understand and apply TCM-related information to promote and maintain good health, which align with the WHO definition of HL and lays a foundation in this domain [33]. Gaining access to TCM-related information refers to the ability to maintain the integrity of TCM, promoting therapeutic understanding, and ensuring patients can access professional health service providers. Understanding TCM-related information refers to individuals’ reading comprehension and numeracy skills which can help them understand health information more optimally. Applying TCM-related information to promote and maintain good health refers to the ability to choose an appropriate self-care method, determine the necessity of seeking professional help, and make positive medical decisions to navigate the dual-medical system. We used the WHO definition to guide our approach to HL as it emphasises a broad understanding of HL that include not only the ability to read and comprehend health information but also the capacity to make informed decisions and take actions based on that information.

Such holistic health education efforts might also benefit the enormous population with chronic pain living in a dual medical system. However, a preliminary literature search revealed that a dearth of research has been conducted regarding TCM-HL in clinical contexts, especially in reference to chronic pain [34]. Questions related to individuals’ access to pain management information or services, knowledge of chronic pain, or ability to self-manage pain and other related issues have been ambiguously investigated. This scoping review aims to expand and more systematically explore this initial literature search by addressing the following questions:

1. What is the conceptual basis underlying the development of health literacy in TCM with respect to chronic pain?
2. What are the TCM-HL measurement tools being used in reference to chronic pain research and practice, and what are their associated psychometric properties?

## Methods

This review follows the methodological approach recommended by Arksey and O’Malley [35]. They recommend that researchers specify the research question, identify relevant literature, summarize, synthesize, and report the results. The systematic search was conducted according to the PRISMA extension for scoping reviews checklist ([36], see Appendix 1 for details).

### Eligibility criteria

Inclusion criteria:

Population: Chinese population, residing in mainland China, Hongkong and Taiwan

Research aims:

- TCM, pain and HL are mentioned;
- Articles should contain at least one aspect of HL (e.g., access to health information or service, understanding of process, diagnosis or treatment, or applying health information or service); and
- Articles should mention how HL has been evaluated

Language: simplified or traditional Chinese, or English

Publication date: no limit-March 2023

Article type: peer reviewed empirical (original research) articles, including randomly controlled trial, cross-sectional study, experimental study or quasi-experimental study.

Exclusion criteria:

- Review article (meta, systematic, scoping), grey literature, commentary, opinion, editorial, book, chapter, thesis, or conference abstract;
- Articles providing no description on how TCM HL was measured;
- Articles where the TCM content is not clearly defined;
- Chronic pain not including persistent attacks (e.g., angina pectoris caused by coronary heart disease, dysmenorrhea); and
- Full text not available.

### Search strategy

We conducted search with five databases: two simplified Chinese (SC) databases (Wanfang and CNKI), one traditional Chinese (TC) database (Airiti Library), and two English databases (PubMed and CINAHL). Wanfang and CNKI were selected because of their popularity among simplified-Chinese-speaking academics. Airiti Library was selected because it included articles published in traditional Chinese which can cover the work from traditional-Chinese-speaking regions such as Taiwan and Hongkong. PubMed and CINAHL were selected to capture relevant articles published in English.

As the research interests were exploring the conceptual model of TCM-HL regarding chronic pain and related measurements, the search terms were focusing on three core elements and categorized according to three groups: Group 1 consisted of terms related to TCM, including different expressions of TCM and their various treatments. Group 2 comprised of terms related to HL, including different expressions of HL. Group 3 contained different expressions of pain (see Table 1). Search results included at least one term from each group (using Boolean Operator ‘OR’ between terms in the same group and ‘AND’ between different groups) (see Appendix 2 for details).

**Table 1:** Search terms in English, simplified Chinese and traditional Chinese

	English	Simplified Chinese	Traditional Chinese
Group 1-TCM	TCM/ Chinese medicine/ traditional medicine/ complementary and alternative medicine/ CAM/ folk medicine/ folk remedy/ acupuncture/ meridian/ acupressure/ massage/ tuina/ Chinese embrocation/ dieda/ ditda/ bone setting/ cupping/ moxibustion/ guasha/ hot compress/ hot pack/ medicated bath/ herbal bath/ foot bath/ foot soak/ reflexology/ auricular/ ear point/ umbilical therapy/ hilum therapy/ fumigation/ qi gong/ daoyin/ tuna/ tai chi/ taiji/ baduanjin/ wuqinxi/ food therapy/ dietary therapy/ Chinese herb/ traditional herb/ herbal medicine/ herbal remedy/ Chinese liniment/ herbal analgesic	中医/传统医学/汉医/养生/治未病/针刺/经络/穴位/指压/点穴/推拿/按摩/跌打/正骨/罐/灸/刮痧/热敷/药浴/足浴/足疗/耳穴/脐疗/敷贴/膏药/熏蒸/气功/导引/吐纳/太极/八段锦/五禽戏/食疗/中药/草药/药酒	中醫/傳統醫學/漢醫/養生/治未病/針刺/經絡/穴位/指壓/點穴/推拿/按摩/跌打/正骨/罐/灸/刮痧/熱敷/藥浴/足浴/足療/耳穴/臍療/敷貼/膏藥/熏蒸/氣功/導引/吐納/太極/八段錦/五禽戲/食療/中藥/草藥/藥酒
Group 2- health literacy	health literacy/ health cultural literacy/ health information literacy/ know believe perform/ health knowledge/ preservation knowledge/ preservation concept/ preservation awareness/ patient intelligence/ health belief/preservation belief/ health concept/ health awareness/ behavior/ behaviour/ health service seeking/ access/ health information access/ understanding/ preference/ decision-making/ communication/ patient empowerment	素养/知信行/健康识能/健康知能/健康认知/健康文化知识/知识获取/健康行为/就医行为/就医选择/医疗服务利用/医病沟通/医患沟通/病人赋权/保健知识/健康观念/健康理念/健康信念/健康意识/保健观念/保健理念/保健信念/保健意识/信息理解/信息获取/知识理解	素養/知信行/健康識能/健康知能/健康認知/健康文化知識/知識獲取/健康行爲/就醫行爲/就醫選擇/醫療服務利用/醫病溝通/醫患溝通/病人賦權/保健知識/健康觀念/健康理念/健康信念/健康意識/保健觀念/保健理念/保健信念/保健意識/信息理解/信息獲取/知識理解
Group 3- pain	pain/ache/sore/hurt/discomfort/distress	痛/炎/酸/不适	痛/炎/酸/不適

As the topic included TCM as the major variable of interest, we chose three language search strategies to cover populations who were most likely familiar with or interested in TCM. Group 1 terms were generated in Chinese and then translated to English. The Chinese search terms were conducted and refined by Chinese-speaking authors and a librarian to generate more partially related terms of HL such as ‘behavior’ or ‘preference’. The key words in Groups 2 and 3 were generated in English and then translated to Chinese. The English search terms were conducted and refined by all authors and a librarian, using both British and US English spelling variations such as ‘behaviour’ and ‘behavior’ to cover more materials for analysis. The traditional Chinese terms were translated from simplified characters which can correspond word by word. The English terms were not the same as some Chinese terms had different expressions in English such as ‘re fu’ translated to ‘hot pack’ or ‘hot compress’, or some English terms had different expressions in Chinese such as ‘health concept’ translates to ‘jian kang guan nian’ or ‘bao jian guan nian’ or ‘jian kang li nian’ or ‘bao jian li nian’. See Table 1 for a summary of the search terms in Chinese and English.

### Selection of sources of evidence

The database search was conducted by the first author, followed by title and abstract screening. Full text screening was conducted by all authors. Articles written in Chinese were checked by the authors YC and GW independently, who are also native Chinese speakers, and English articles were reviewed by MH, a native English speaker. The included articles needed to achieve consensus between authors according to the inclusion and exclusion criteria.

### Data charting process and synthesis of results

All authors discussed and reached an agreement on which information should be extracted and summarised, the information extracted and included in the final analysis were discussed and determined by all authors. Extracted data consisted of original information including settings (i.e., community health centre, tertiary hospital, or clinic), types of pain, research designs, participant details, and TCM-HL-relevant measures have been explored (see Table 2). We adopted the WHO’s definition to identify elements to conceptualize HL or items to measure HL in three categories to access, understand and apply health information to manage chronic pain using TCM.

**Table 2:** Information of included articles

**Author, Year, Setting**	**Type of pain**	**Research design, participant details**	**HL focus**	**Key items used in the measure**	**Psychometric properties**	**Elements of HL**
Feng et.al., 2007 [37] community centre	lumbar and limb chronic pain	RCT (n= 68), 50~78 yrs., and 42% female	Mastery level of self-preservation knowledge	No items shown but a mastery score was presented	Not validated	Apply
Liang et.al., 2008 [38] tertiary hospital	neck, shoulder, lumbar, and leg pain	Cross-sectional (n=100), 16~74 yrs., and 42% female	Awareness of hot compress	8 items: how patients understand the usage, placement and effect of hot compress (the hotter the medicine pack, the better the effect; the longer the heat compress applied, the better the effect was; the more frequent heat compresses, the better the effect; place the medicine bag on the lumbar or back in the prone position; put pack in butts or legs and other pain areas; wrap the medicine bag with cloth in many layers; wrap the medicine bag in plastic bag; hot compress is not important, it can be applied or not applied)	Not validated	Understand
Huang et. al., 2009 [39] tertiary hospital	lumbar intervertebral disc herniation	RCT (n=68), 43~73 yrs., and 44% female	Mastery level of diet, exercise, TCM method, health awareness and emotion knowledge	No items shown but mastery scores were presented	Not validated	Apply
Li, 2010 [40] tertiary hospital	osteoporosis	RCT (n=112), 60~97 yrs., and 44% female	Mastery level of diet, TCM method, health awareness and emotion knowledge	No items shown but mastery scores were presented	Not validated	Apply
Sun et.al., 2011 [41] community centre	osteoporosis; neck, shoulder, lumbar, and leg pain; arthritic rehabilitation	Cross-sectional (n=391), 45~59 yrs., and 50% female	Mastery level of disease-related TCM preservation or treatment knowledge	No items shown but mastery scores were presented	Cronbach's α 0.955	Apply
Cao, & Qiu, 2011 [42] tertiary hospital	knee osteoarthritis	RCT (n=60), age (NA), and gender (NA)	Mastery level of KOA knowledge	No items shown but a mastery score was presented	Not validated	Apply
Li, 2012 [43] community centre	rheumatoid arthritis	Quasi-experimental (n=105), 50±16 yrs., and 76% female	Mastery level of TCM and western medicine nursing knowledge	No items shown but a mastery score was presented	Not validated	Apply
Yue, & Yang, 2012 [44] community centre	general TCM knowledge with statements related to measure pain management ability	Cross-sectional (n=208), 47% 30~40 yrs, and gender (NA)	Mastery level of basic concepts, Chinese medicine knowledge, diet and diagnosis terms, attitude towards TCM knowledge, and TCM healthcare-seeking behaviour	25 items: community nurses’ understanding of TCM treatments, perceptions of TCM 18 items: attitude towards TCM, 1 item related to pain 20 items: TCM experience for pain management, 2 items related to pain	Internal consistency Face validity	Understand Apply
Guan, 2013 [45] tertiary hospital	lumbar disc protrusion	RCT (n=70), 43~73 yrs., and 44% female	Mastery level of TCM preservation method	No items shown but a mastery score was presented	Not validated	Apply
Yang, & Chen, 2013 [46] community centre	osteoarthritis	Cross-sectional (n=388), 41~72 yrs., and 54% female	Mastery level of basic knowledge and osteoarthritis-related risks	No item details shown but one statement related: practise the horse-riding squat as an exercise for OA	Not validated	Apply
Chen et.al., 2015 [47] tertiary hospital	low back pain	Cross-sectional (n=101), 22~74 yrs., and 76% female	Understand level of treatment sensation	2 items: patients understanding of sensations during the acupuncture treatment (‘sore and numb’ or ‘sense of mild thermal and vibration’)	Not validated	Understand
Zheng et.al., 2015 [48] tertiary hospital	knee osteoarthritis	Cross-sectional (n=148), 88% female	TCM treatments selection rate	No item details shown but one statement related: patients’ or practitioners’ choice of TCM treatments	Not validated	Apply
Yuan, 2015 [49] community centre	osteoporosis; neck, shoulder, lumbar, and leg pain; arthritic rehabilitation	Quasi-experimental (n=399), 60~78 yrs., and 43% female	Mastery level of TCM dietary guidance and disease-related TCM preservation or treatment knowledge	No items shown but mastery scores were presented	Not validated	Apply
Jin, & Qiu, 2015 [50] community centre	general TCM knowledge and attitude with statements related to measure pain management ability	Cross-sectional (n=1726), 47.24±15.28 yrs., and 65% female	Awareness and utilization of TCM health service	6 items: residents’ perspectives towards TCM health service, 1 item related to pain	Cronbach's α 0.833 KMO 0.715 Bartlett	Understand
Zheng et.al., 2017 [51] tertiary hospital	general TCM knowledge and attitude with statements related to measure pain management ability	Cross-sectional (n=218), age (NA), and gender (NA)	Problems of the clinical application of TCM nursing therapies	No items shown but a mastery score was presented	Cronbach's α 0.83 Content validity: CVI 0.87	Apply
He et.al., 2017 [52] community centre	knee osteoarthritis	Cross-sectional (n=1000), 40~82 yrs., and 55% female	Know, believe and perform rate of TCM fumigation Sources of health information	No items shown but rates were presented 1 item: choice of information sources	Not validated	Access Apply
Liu, 2017 [53] tertiary hospital	lumbar intervertebral disc herniation	RCT (n=40), 36~70 yrs., and 30% female	Mastery level of diet, TCM method, health awareness and emotion knowledge	No items shown but mastery scores were presented	Not validated	Apply
Zen et.al., 2017 [54] tertiary hospital	osteoporosis	RCT (n=86), 56~81 yrs., and 33% female	Mastery level of osteoporosis knowledge	No items shown but a mastery score was presented	Not validated	Apply
Pang et.al., 2019 [55] community centre	knee osteoarthritis	RCT (n=60), 38~64 yrs., and 73% female	Mastery level of disease and self-care knowledge	No items shown but a mastery score was presented	Not validated	Apply
Li et.al., 2019 [56] Tertiary hospital	knee osteoarthritis	Cross-sectional (n=162), 18~80+ yrs., and 61% female	Mastery level of KOA diagnosis and treatment knowledge and physical exercise knowledge	13 items: 2 items related to TCM (KOA can be treated by TCM methods; practise the horse-riding squat as an exercise for KOA)	Not validated	Apply
Zha, 2019 [57] community centre	Osteoporosis; neck, shoulder, lumbar, and leg pain; arthritis; rehabilitation	Quasi-experimental (n=56), 61~77 yrs., and 46% female	Mastery level of general TCM preservation knowledge and disease-related TCM preservation knowledge	No items shown but mastery scores were presented	Not validated	Apply
Zeng, et.al., 2020 [58] tertiary hospital	knee osteoarthritis	Delphi (n=71), and 47% female	Importance level of mastering knowledge of preventing exogenous and preserving methods sources of self-management and TCM health information	13 items related to knowledge of preventing and self-managing disease and sources of health education information are assessed by experts	Content validity	Access Apply
Hu, 2020 [59] tertiary hospital	knee osteoarthritis	RCT (n=76), 45~80 yrs., and 61% female	Implementation rate of diet, precaution and exercise	No items shown but rates were presented	Not validated	Apply
Chen, & Yan, 2021 [60] clinic	soft tissue injury	Cross-sectional (n=254), 45~75+ yrs., and 65.2% female	Awareness of the procedure of acupuncture	9 items: Before treatments: Acupuncture points are disinfected with alcohol cotton balls before acupuncture; Appropriate postures will be designated before acupuncture to facilitate acupoint selection; Acupuncture uses disposable filigree needles and there is no risk of infection. During treatments: Acupuncture points will feel sore and numb. Do not move your body arbitrarily during acupuncture; If you are too hungry, nervous, weak, or drink alcohol, you are prone to dizziness, nausea, cold sweats, chest tightness and other dizziness; You should relax your mind during acupuncture, otherwise it is easy to have the illusion of acupuncture pain. After treatments: If there is bleeding at the acupuncture point when the needle is pulled out, the medical staff will press the cotton ball to stop the bleeding; If acupuncture points are bruised, they will return to normal within a few days to two weeks; Acupuncture can be performed concurrently with other treatments (e.g., rehabilitation, massage).	Cronbach's α 0.839 Content validity (no data)	Understand
Lan, et.al., 2021 [61] tertiary hospital	knee osteoarthritis	RCT (n=86), 55~83 yrs., and 56% female	Mastery level of KOA and Zhuang herb mud moxibustion knowledge	No items shown but mastery scores were presented	Not validated	Apply
Niu et. al., 2021 [62] tertiary hospital	knee osteoarthritis	Delphi (n=15), 30~50 yrs, and gender (NA).	Mastery level of TCM method, differentiation and diet, post-discharge preservation knowledge	No items shown but mastery scores were presented	Face validity	Apply
Fei, 2021 [63] tertiary hospital	rheumatoid arthritis	RCT (n=62), 39~76 yrs., and 39% female	Mastery level of health knowledge	No items shown but a mastery score was presented	Not validated	Apply
Liu et.al., 2022 [64] clinic	chronic musculoskeletal (MSK) pain	Cross-sectional (n=55), 65~90+ yrs., and 69.1% female	Awareness of Chinese medicine	3 items: source of health information; Chinese medicine treatments were believed to be benefit; Chinese medicine treatments for coping with MSK	Not validated	Access Understand Apply

## Results

### Selection of source of evidence

**Fig. 1:**
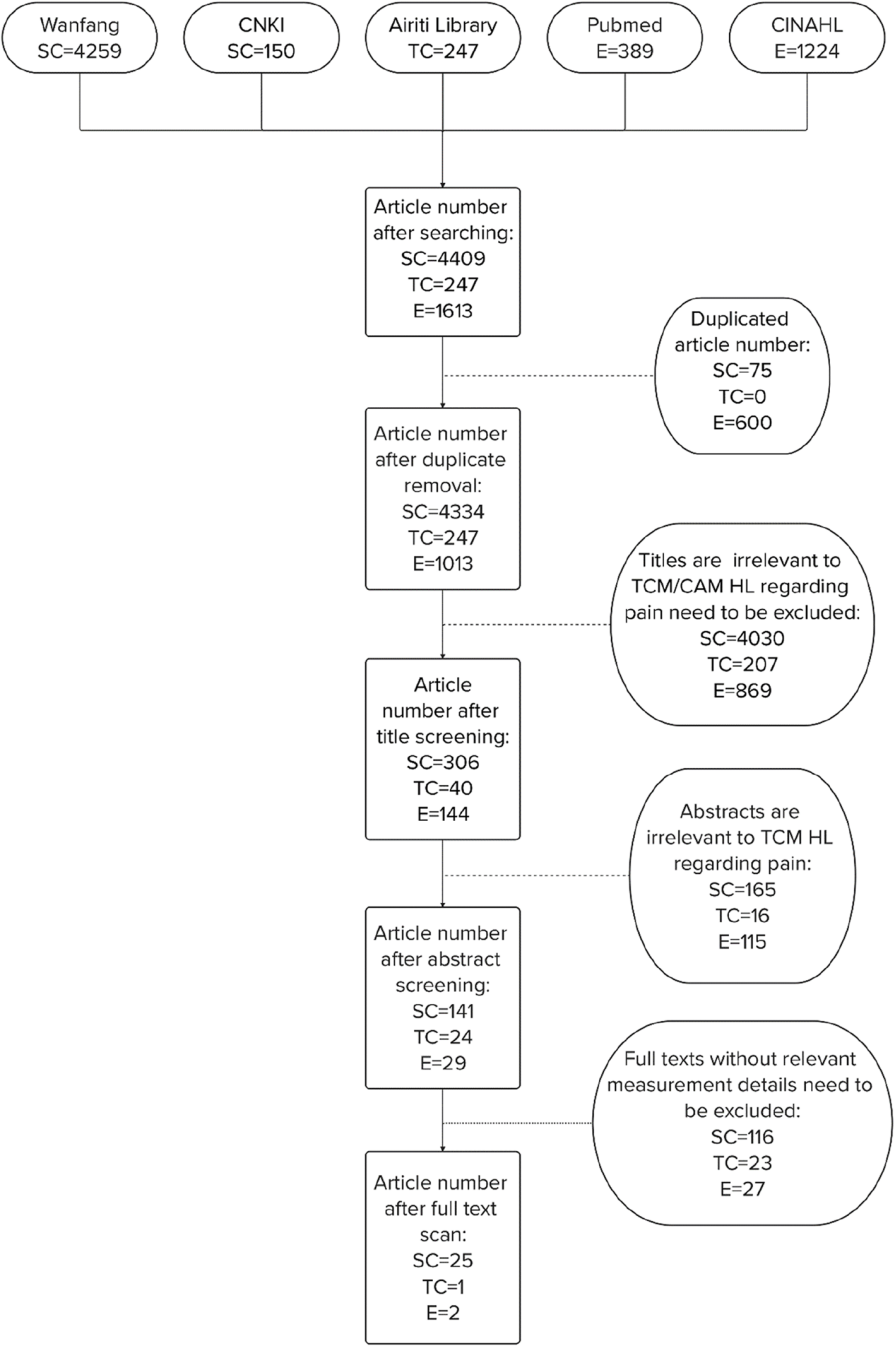
Searches and screening flow according to PRISMA-ScR. SC: simplified Chinese article; TC: traditional Chinese article; E: English article

The first search phase generated 4409 simplified Chinese articles, 247 traditional Chinese articles, and 1613 English articles. After deleting duplications, 5594 articles remained. During the title screening stage, 5106 articles were excluded as their titles were not related to TCM or CAM (complementary and alternative medicine) HL regarding pain. During the abstract screening stage, 296 articles were excluded as their abstracts revealed no content related to TCM HL regarding pain. During the full text screening stage, 166 articles were excluded as there were no measurement or description detail related to TCM HL regarding pain. Twenty-five simplified Chinese articles, one traditional Chinese article, and two English articles were included for the final review. The PRISMA-ScR flow diagram is shown in Fig. 1.

### Measurement tools in reference to TCM health literacy and chronic pain

The publication years of selected articles ranged from 2007 to 2022. Three studies were conducted in Taiwan [47, 60, 64], while others originated from mainland China. The majority of the studies (16 out of 28, 57%) were conducted in tertiary hospitals and the remaining studies were coordinated in either community hospitals or private clinics (see Table 2). Most studies (19 out of 28, 68%) measured the TCM health literacy level with knowledge mastery scores with or without education. Six studies reported the understanding level of certain diseases or treatments as a reflection of health literacy level, two articles mentioned the selection rate of certain treatments or diets, and one article explored the agreement of importance of health literacy items.

Various disease types and research methods were included in the articles. The most commonly studied disease was osteoarthritis (OA, N = 11), nine of which were knee osteoarthritis (KOA). There were nine articles concentrating on lumbar pain, one focusing on lower back pain (LBP), three focusing on lumbar disc herniation, one related to lumbar and leg regions, and four articles investigated pain associated with joint pain in the neck, shoulder, lumbar, and leg. Five articles studied osteoporosis, among which two focused specifically on osteoporosis and three mentioned osteoporosis along with other chronic pain diseases. Arthritis, rehabilitation and general TCM knowledge with statements related to measure pain management ability were studied in three articles, respectively. Two articles focused on rheumatoid arthritis and one article studied soft tissue injury. The most commonly employed research designs were cross-sectional studies (n=12) and randomized controlled trials (RCT, n=11). Three studies used quasi-experimental methods and two used a Delphi method (see Table 2).

### Psychometric quality of measurement tools

There was a scarcity of item details presented in the reviewed articles and only a few of them reported reliability and validity measures. Seven out of the 28 included articles contained information on the psychometric properties of the measures employed, while the remaining 21 articles (seven cross-sectional studies, three quasi-experimental studies and 11 RCTs) did not present any psychometric details, making it difficult to appraise their evidence quality. Cronbach's α was the most frequently used statistical method to measure reliability and item internal consistency of the item sets used within the questionnaires. One article [44] described its reliability with a statement of ‘internal consistency’, but it was not clear which method they used (e.g., Cronbach's α, split-half, or test-retest). To reflect the validity of the measurements, content validity was the most commonly used method (n = 5). Two Delphi studies [58, 62] reported expert authority coefficients and coordination coefficients. One cross-sectional study [51] presented the content validity index (CVI) to show acceptable content validity, and another article mentioned face validity by experts but no further details were presented [60]. Commonly used exploratory factor analysis measures, such as KMO (Kaiser-Meyer-Olkin) and Bartlett, were used in one article [50] to determine construct validity (see Table 2). Most articles stated increases in scores after health education with ambiguous item and psychometric details.

### TCM-HL conceptual basis and application in chronic pain area

As seen in Table 2, the reviewed measurement tools had a different theoretical basis underlying health literacy as a concept. Only one article [64] covered all three aspects of the WHO HL framework (as aforementioned) referring to: apply (how health literacy can be applied in clinical and community settings), understand (how health literacy is understood in populations) and access (how health literacy information can be accessed). The remaining twenty-seven articles conceptualized and measured TCM-HL according to one or two of these aspects. Twenty-four out of 28 studies mentioned the ‘apply’ construct with or without other facets, among which 20 focused only on the ‘apply’ facet. The most frequently adopted method to measure the ‘apply’ facet occurred when comparing participants’ scores on level of knowledge mastery of certain diseases before and after health education. Twelve articles mentioned the knowledge level related to self-preservation methods, and six articles mentioned the knowledge level of certain disease. For example, Li [40] reported that participants exhibited enhanced knowledge of diet, exercise, TCM methods, health awareness and emotional wellbeing after receiving an educational intervention from nurses regarding constitution differentiation, personalized diets and self-monitor strategies. Six articles explored the ‘understand’ facet by investigating patients’ awareness of TCM treatment procedures or outcomes such as acupuncture sensation [47] or hot compress therapy [38]. The ‘access’ facet was explored in three articles by investigating how people engaged in self-management and employing TCM health education information [52, 58, 64]. Therefore, the conceptual basis of existing relevant studies was not conclusive which creates a problem of inferring a meaningful perspective and might also result in assessment inaccuracies.

## Discussion

Our findings indicated that the conceptual basis of existing measures of TCM-HL regarding chronic pain fails to correspond to all three facets of the WHO’s definition, which is the ability to gain access, understand and apply health information or service to maintain a healthy status [13]. Only a few papers clarified the definition or conceptual model of the TCM-HL before developing a measurement tool. The majority of these tools only explored one or two facets of health literacy and none of the reviewed tools measured people’s ability to navigate the dual medical system. The psychometric quality of measurement tools was often difficult to discern. However, a few studies have shown some evidence of reliability and validity, while most of the included articles failed to conduct an adequate psychometric evaluation. This lack of rigorous transparency makes the assessment of measurement tools difficult and the statement of results less defensible. A lack of emphasis on a comprehensive conceptual model for TCM-HL or limited cultural adaptation of tools that were developed in the western medical system may have contributed to the design and development of culturally inappropriate measurement tools [26].

In the current review, only one measurement tool [64] captured all three aspects of the WHO HL framework, but details regarding the item domains were limited. For example, to assess the ‘understand’ facet, only a single item was used to measure the percentage of those people believing the effectiveness of Chinese medicine treatments among rural older adults with chronic musculoskeletal pain in Taiwan. However, this single item could only reflect patients’ subjective opinions and was not able to accurately measure the targeted ability level because only a few validated items were being used. Furthermore, in most of the reviewed articles, the coverage of health literacy was limited to the mastery level of TCM preservation knowledge. TCM health literacy is usually evaluated with a supplementary measurement tool to attest the secondary benefit of health education during treatments. It is important to note that both practitioners and researchers play a role in promoting health literacy, which in turn supports pain management and leads to better health outcomes [15].

Multiple factors have contributed to a lack of quality-assured research that evaluates the efficacy of TCM health literacy in the remediation of chronic pain. Firstly, practitioners in China could be key agents in promoting TCM health literacy. However, they may not be trained health ‘educators’ and their primary task is providing healthcare to large numbers of patients waiting for immediate diagnosis and prescription [65]. Secondly, researchers need to be instrumental in developing valid and reliable measurement tools. However, they have not managed this requirement in the resulting research outputs, and this may be due to conflicts between teaching, research and clinical duties. As such, developing effective treatments/cures are prioritized in the clinical context instead of exploring ways to understand and promote patients’ health literacy skills. Thirdly, the large population in China means that there is a huge variation in people’s health literacy levels, and this poses challenges to the healthcare system. Both health education and healthcare systems should be involved to promote the population’s health literacy levels and self-management skills rather than relying on a practitioners’ one-sided healthcare approach (TCM or western medicine), which is likely to be inadequate. However, this could be difficult to achieve due to limited resources. The purpose of developing health literacy is to improve patients’ communication and self-care skills as well as optimizing the medical system [66, 67], therefore, more emphasis should be put on catering to patients’ needs when considering long-term population health. The Chinese government has realized the importance of HL by publishing the official TCM-HL questionnaire [30]. The exploration of TCM health literacy in the chronic pain area can contribute to both individuals and the health system by promoting self-management and more equitable distribution of health resources. For example, the misunderstanding of the use of a hot compress might influence a patient’s medical decision by overusing it in an inappropriate manner or totally rejecting this treatment even if it is a cost-effective method.

There are several limitations associated with this review. One is related to the development of search terms. Although efforts were made to cover all available articles in the area, it is possible that some studies were missed due to different pain-related expressions or conditions which were not directly related to pain but have measured health literacy. Another issue is related to the exclusion process, given that all reviewed studies were focussed on Chinese populations in mainland China, Hong Kong, and Taiwan. The results might be more comprehensive and applicable if the review had included other regions with Chinese cultural backgrounds. We used the WHO’s definition of HL to assess the conceptual basis of existing measures. This may have limited our ability to detect other domains of HL. The strength of the study is in providing a clear view of the current conceptual basis of TCM-HL and existing measurement tools, which can be a foundation for developing more culturally-specific assessment models and methods.

Future studies could further explore how TCM health literacy could benefit public health. For the Chinese population or other TCM consumers, focus should be on developing more comprehensive measurement tools to accurately assess TCM health literacy level, as well as improving health education programmes. In addition, conducting robust research to develop valid and reliable measurement tools could provide more evidence on how TCM health literacy could contribute to improving population health outcomes. For example, chronic pain populations with higher TCM health literacy level will have better abilities in regard to obtaining related information, understanding essential knowledge, applying appropriate methods, and navigating between different medical systems, which benefit both individual pain management and public health.

## Conclusion

Current research reflects some deficiencies regarding how TCM HL has been studied in the field of chronic pain. These deficiencies include the underestimation of its importance, the ambiguous nature when defining concepts, and the overabundance of low-quality measurements. It is proposed that future research should focus on exploring the development of clearer definitions, advancing more comprehensive conceptual frameworks, and actively engaging in the design and implementation of high-quality, psychometrically robust assessment tools.

## Data Availability

All data generated or analysed during this study are derived from journal articles available in the following public databases: PubMed https://pubmed.ncbi.nlm.nih.gov CINAHL https://www.ebsco.com/products/research-databases/cinahl-database CNKI https://www.cnki.net Wanfang https://www.wanfangdata.com.cn Airiti Library https://www.airitilibrary.com

## Appendix 1

Preferred Reporting Items for Systematic reviews and Meta-Analyses extension for Scoping Reviews (PRISMA-ScR) Checklist

**Table:**

**SECTION**	**ITEM**	**PRISMA-ScR CHECKLIST ITEM**	**REPORTED ON PAGE #**
**TITLE**			
Title	1	Identify the report as a scoping review.	1
**ABSTRACT**			
Structured summary	2	Provide a structured summary that includes (as applicable): background, objectives, eligibility criteria, sources of evidence, charting methods, results, and conclusions that relate to the review questions and objectives.	1,2
**INTRODUCTION**			
Rationale	3	Describe the rationale for the review in the context of what is already known. Explain why the review questions/objectives lend themselves to a scoping review approach.	3-5
Objectives	4	Provide an explicit statement of the questions and objectives being addressed with reference to their key elements (e.g., population or participants, concepts, and context) or other relevant key elements used to conceptualize the review questions and/or objectives.	5
**METHODS**			
Protocol and registration	5	Indicate whether a review protocol exists; state if and where it can be accessed (e.g., a Web address); and if available, provide registration information, including the registration number.	NA
Eligibility criteria	6	Specify characteristics of the sources of evidence used as eligibility criteria (e.g., years considered, language, and publication status), and provide a rationale.	6
Information sources^a^	7	Describe all information sources in the search (e.g., databases with dates of coverage and contact with authors to identify additional sources), as well as the date the most recent search was executed.	6,7
Search	8	Present the full electronic search strategy for at least 1 database, including any limits used, such that it could be repeated.	7
Selection of sources of evidence^b^	9	State the process for selecting sources of evidence (i.e., screening and eligibility) included in the scoping review.	7,8
Data charting process^c^	10	Describe the methods of charting data from the included sources of evidence (e.g., calibrated forms or forms that have been tested by the team before their use, and whether data charting was done independently or in duplicate) and any processes for obtaining and confirming data from investigators.	8
Data items	11	List and define all variables for which data were sought and any assumptions and simplifications made.	7
Critical appraisal of individual sources of evidence^d^	12	If done, provide a rationale for conducting a critical appraisal of included sources of evidence; describe the methods used and how this information was used in any data synthesis (if appropriate).	NA
Synthesis of results	13	Describe the methods of handling and summarizing the data that were charted.	8
**RESULTS**			
Selection of sources of evidence	14	Give numbers of sources of evidence screened, assessed for eligibility, and included in the review, with reasons for exclusions at each stage, ideally using a flow diagram.	8
Characteristics of sources of evidence	15	For each source of evidence, present characteristics for which data were charted and provide the citations.	8,9
Critical appraisal within sources of evidence	16	If done, present data on critical appraisal of included sources of evidence (see item 12).	NA
Results of individual sources of evidence	17	For each included source of evidence, present the relevant data that were charted that relate to the review questions and objectives.	9,10
Synthesis of results	18	Summarize and/or present the charting results as they relate to the review questions and objectives.	9,10
**DISCUSSION**			
Summary of evidence	19	Summarize the main results (including an overview of concepts, themes, and types of evidence available), link to the review questions and objectives, and consider the relevance to key groups.	10-12
Limitations	20	Discuss the limitations of the scoping review process.	13-14
Conclusions	21	Provide a general interpretation of the results with respect to the review questions and objectives, as well as potential implications and/or next steps.	14
**FUNDING**			
Funding	22	Describe sources of funding for the included sources of evidence, as well as sources of funding for the scoping review. Describe the role of the funders of the scoping review.	31

*JBI* Joanna Briggs Institute, *PRISMA-ScR* Preferred Reporting Items for Systematic reviews and Meta-Analyses extension for Scoping Reviews

^a^Where *sources of evidence* (see second footnote) are compiled from, such as bibliographic databases, social media platforms, and Web sites

^b^A more inclusive/heterogeneous term used to account for the different types of evidence or data sources (e.g., quantitative and/or qualitative research, expert opinion, and policy documents) that may be eligible in a scoping review as opposed to only studies. This is not to be confused with *information sources* (see first footnote)

^c^The frameworks by Arksey and O’Malley [6] and Levac and colleagues [7] and the JBI guidance [4, 5] refer to the process of data extraction in a scoping review as data charting

^d^The process of systematically examining research evidence to assess its validity, results, and relevance before using it to inform a decision. This term is used for items 12 and 19 instead of "risk of bias" (which is more applicable to systematic reviews of interventions) to include and acknowledge the various sources of evidence that may be used in a scoping review (e.g., quantitative and/or qualitative research, expert opinion, and policy document)

*From:* Tricco AC, Lillie E, Zarin W, O'Brien KK, Colquhoun H, Levac D, et al. PRISMA Extension for Scoping Reviews (PRISMAScR): Checklist and Explanation. Ann Intern Med. 2018;169:467–473. 10.7326/M18-0850.

## Appendix 2

Search strategy in five databases

**Table:**

**Database**	**Inputs in advanced searching box**
Wanfang	主题:(中医 or 传统医学 or 汉医 or 养生 or 治未病 or 针刺 or 经络 or 穴位 or 指压 or 点穴 or 推拿 or 按摩 or 跌打 or 正骨 or 罐 or 灸 or 刮痧 or 热敷 or 药浴 or 足浴 or 足疗 or 耳穴 or 脐疗 or 敷贴 or 膏药 or 熏蒸 or 气功 or 导引 or 吐纳 or 太极 or 八段锦 or 五禽戏 or 食疗 or 中药 or 草药 or 药酒) and 主题:(素养 or 知信行 or 健康识能 or 健康知能 or 健康认知 or 健康认识 or 健康文化知识 or 知识获取 or 健康行为 or 就医行为 or 信息理解 or 就医选择 or 医疗服务利用 or 沟通 or 病人赋权 or 保健知识 or 健康观念 or 健康理念 or 健康信念 or 健康意识 or 保健观念 or 保健理念 or 保健信念 or 保健意识) and 主题:(痛 or 炎 or 酸 or 不适)
CNKI	TKA=(中医 + 传统医学 + 汉医 + 养生 + 治未病 + 针刺 + 经络 + 穴位 + 指压 + 点穴 + 推拿 + 按摩 + 跌打 + 正骨 + 罐 + 灸 + 刮痧 + 热敷 + 药浴 + 足浴 + 足疗 + 耳穴 + 脐疗 + 敷贴 + 膏药 + 熏蒸 + 气功 + 导引 + 吐纳 + 太极 + 八段锦 + 五禽戏 + 食疗 + 中药 + 草药 + 药酒) and (素养 + 知信行 + 健康识能 + 健康知能 + 健康认知 + 健康文化知识 + 知识获取 + 健康行为 + 就医行为 + 信息理解 + 就医选择 + 医疗服务利用 + 沟通 + 病人赋权 + 保健知识 + 健康观念 + 保健观念 + 保健理念 + 健康信念 + 保健信念 + 健康理念 + 健康意识 + 保健意识) and (痛 + 炎 + 酸 + 不适)
Airiti Library	(中醫 OR 傳統醫學 OR 漢醫 OR 養生 OR 治未病 OR 針刺 OR 經絡 OR 穴位 OR 指壓 OR 點穴 OR 推拿 OR 按摩 OR 跌打 OR 正骨 OR 罐 OR 灸 OR 刮痧 OR 熱敷 OR 藥浴 OR 足浴 OR 耳穴 OR 臍療 OR 敷貼 OR 膏藥 OR 熏蒸 OR 氣功 OR 導引 OR 吐納 OR 太極 OR 八段錦 OR 五禽戲 OR 食療 OR 中藥 OR 草藥 OR 藥酒) AND (素養 OR 知信行 OR 健康識能 OR 健康知能 OR 健康認知 OR 健康文化知識 OR 知識獲取 OR 健康行爲 OR 就醫行爲 OR 信息理解 OR 就醫選擇 OR 醫療服務利用 OR 溝通 OR 病人賦權 OR 保健知識 OR 健康觀念 OR 保健觀念 OR 保健理念 OR 健康信念 OR 保健信念 OR 健康理念 OR 健康意識 OR 保健意識) AND (痛 OR 炎 OR 酸 OR 不適)
Pubmed	((TCM[Title/Abstract]) OR (Chinese medicine[Title/Abstract]) OR (traditional medicine[Title/Abstract]) OR (complementary and alternative medicine[Title/Abstract]) OR (CAM[Title/Abstract]) OR (folk medicine[Title/Abstract]) OR (folk remedy[Title/Abstract]) OR (acupuncture[Title/Abstract]) OR (meridian[Title/Abstract]) OR (acupressure[Title/Abstract]) OR(massage[Title/Abstract]) OR (tuina[Title/Abstract]) OR (Chinese embrocation[Title/Abstract]) OR (dieda[Title/Abstract]) OR (ditda[Title/Abstract]) OR (bone setting[Title/Abstract]) OR (cupping[Title/Abstract]) OR (moxibustion[Title/Abstract]) OR (guasha[Title/Abstract]) OR (hot compress[Title/Abstract]) OR (hot pack[Title/Abstract]) OR (medicated bath[Title/Abstract]) OR (herbal bath[Title/Abstract]) OR (foot bath[Title/Abstract]) OR (foot soak[Title/Abstract]) OR (auricular[Title/Abstract]) OR (ear point[Title/Abstract]) OR (umbilical therapy[Title/Abstract]) OR (hilum therapy[Title/Abstract]) OR (fumigation[Title/Abstract]) OR (reflexology[Title/Abstract]) OR (qi gong[Title/Abstract]) OR (daoyin[Title/Abstract]) OR (tuna[Title/Abstract]) OR (tai chi[Title/Abstract]) OR (taiji[Title/Abstract]) OR (baduanjin[Title/Abstract]) OR (wuqinxi[Title/Abstract]) OR (food therapy[Title/Abstract]) OR (dietary therapy[Title/Abstract]) OR (Chinese herb[Title/Abstract]) OR (traditional herb[Title/Abstract]) OR (herbal medicine[Title/Abstract]) OR (Chinese liniment[Title/Abstract]) OR (herbal analgesic[Title/Abstract])) AND ((health literacy[Title/Abstract]) OR (health cultural literacy[Title/Abstract]) OR (health information literacy[Title/Abstract]) OR (know believe perform[Title/Abstract]) OR (health knowledge[Title/Abstract]) OR (preservation knowledge[Title/Abstract]) OR (preservation concept[Title/Abstract]) OR (preservation awareness[Title/Abstract]) OR (patient intelligence[Title/Abstract]) OR (health belief[Title/Abstract]) OR (preservation belief[Title/Abstract]) OR (health concept[Title/Abstract]) OR (health awareness[Title/Abstract]) OR (behavior[Title/Abstract]) OR (health service seeking[Title/Abstract]) OR (access[Title/Abstract]) OR (behaviour[Title/Abstract]) OR (understanding[Title/Abstract]) OR (preference[Title/Abstract]) OR (decision-making[Title/Abstract]) OR (communication[Title/Abstract]) OR (patient empowerment[Title/Abstract])) AND ((pain[Title/Abstract]) OR (ache[Title/Abstract]) OR (sore[Title/Abstract]) OR (hurt[Title/Abstract]) OR (discomfort[Title/Abstract]) OR (distress[Title/Abstract]))
CINAHL	TCM OR Chinese medicine OR traditional medicine OR complementary and alternative medicine OR CAM OR folk medicine OR folk remedy OR acupuncture OR meridian OR acupressure OR massage OR tuina OR Chinese embrocation OR dieda OR ditda OR bone setting OR cupping OR moxibustion OR guasha OR hot compress OR hot pack OR medicated bath OR herbal bath OR foot bath OR foot soak OR reflexology OR auricular OR ear point OR umbilical therapy OR hilum therapy OR fumigation OR qi gong OR daoyin OR tuna OR tai chi OR taiji OR baduanjin OR wuqinxi OR food therapy OR dietary therapy OR Chinese herb OR traditional herb OR herbal medicine OR herbal remedy OR Chinese liniment OR herbal analgesic health literacy OR health cultural literacy OR health information literacy OR know believe perform/health knowledge OR preservation knowledge OR preservation concept OR preservation awareness OR patient intelligence OR health belief OR preservation belief OR health concept OR health awareness OR behavior OR behaviour OR health service seeking OR access OR health information access OR understanding OR preference OR decision-making OR communication OR patient empowerment pain OR ache OR sore OR hurt OR discomfort OR distress

## Abbreviations

BHLS: Brief Health Literacy Screen
CAM: Complementary and alternative medicine
CVI: Content validity index
HL: Health literacy
KMO: Kaiser-Meyer-Olkin
KOA: Knee osteoarthritis
LBP: Lower back pain
NVS: Newest Vital Sign
NA: Not applicable
OA: Osteoarthritis
RCT: Randomized controlled trials
REALM: Rapid Estimate of Adult Literacy in Medicine
SC: Simplified Chinese
SILS: Single Item Literacy Screener
S-TOFHLA: Short Test of Functional HL in Adults
TC: Traditional Chinese
TCM: Traditional Chinese medicine
